# What is the effect of patient-reported outcome (PRO) item order on prioritisation of PROs in the development of a core outcome set?

**DOI:** 10.1186/1745-6215-16-S2-O73

**Published:** 2015-11-16

**Authors:** Katy Chalmers, Kerry Avery, Karen Coulman, Natalie Blencowe, Rhiannon Macefield, Chris Metcalfe, Jane Blazeby, Sara Brookes

**Affiliations:** 1 University of Bristol, Bristol, UK

## Background

Core outcome sets (COS) are a minimum set of outcomes to be measured and reported in all trials of a specific condition. Questionnaires are often used in their development to enable stakeholders to rate potential outcomes in terms of importance. This study examined the impact of the ordering of patient-reported outcomes (PRO) within the questionnaire on their prioritisation.

## Methods

The order of questionnaire items in the development of a COS for oesophageal cancer surgery was randomised between PROs appearing first (V1) and last (V2). Comparisons were made between the percentages of items rated as essential (scored 7-9 by ≥70%) in the two versions.

## Results

115 patients and 68 healthcare professionals completed questionnaires (98 and 85 randomised to V1 and V2 respectively). The percentage of PRO items rated as essential in V1 was 31.6% and in V2 63.2% (difference=31.6%, 95% CI 14.2-49.0, P<0.001). Looking at stakeholder groups separately, patients rated 36.8% essential in V1 and 78.9% in V2 (42.1%, 95% CI 23.8-60.4, P<0.0001) and professionals 31.6% in V1 and 18.4% in V2 (13.2%, 95% CI -30.7-4.4, P=0.096).

## Conclusion

The order of PRO items (first or last) in the questionnaire is important and the impact of position is dependent on stakeholder group. Patients are more likely to rate PRO items as essential when they appear last in the questionnaire, whereas professionals are more likely to rate them as essential when they appear first. Qualitative studies need to be done to try and understand why this remarkable difference in PRO scoring occurs.

